# Preventive efficacy of six monthly oral doses of Simparica Trio^®^, Heartgard^®^ Plus, and Interceptor^®^ Plus against a macrocyclic lactone-resistant strain (ZoeLA) of heartworm (*Dirofilaria immitis*) in dogs

**DOI:** 10.1186/s13071-022-05180-3

**Published:** 2022-03-11

**Authors:** Jamie A. E. Myers, Susan Holzmer, John W. McCall, Sean P. Mahabir, Tom L. McTier, Steven J. Maeder, Kristina Kryda

**Affiliations:** 1 Zoetis, Veterinary Medicine Research and Development, 333 Portage Street, Kalamazoo, MI 49007 USA; 2 TRS Labs Inc, 215 Paradise Blvd, Athens, GA 30607 USA

**Keywords:** Canine, *Dirofilaria immitis*, Heartworm, Simparica Trio^®^, Heartgard^®^ Plus, Interceptor^®^ Plus, Laboratory study, Macrocyclic lactone, Moxidectin, Resistance

## Abstract

**Background:**

Administration of four to six consecutive monthly doses of 24 µg/kg moxidectin alone shows high effectiveness in preventing the maturation of macrocyclic lactone (ML)-resistant heartworm strains, *Dirofilaria immitis* JYD-34 and ZoeLA. This laboratory study evaluated the efficacy of six consecutive monthly oral doses of Simparica Trio^®^ (moxidectin/sarolaner/pyrantel) compared to six monthly doses of either Heartgard^®^ Plus (ivermectin/pyrantel) or Interceptor^®^ Plus (milbemycin oxime/praziquantel) against ML-resistant *D. immitis* ZoeLA strain.

**Methods:**

Beagle dogs were inoculated with 50 third-stage (L3) *D. immitis* larvae (ZoeLA) 30 days prior to the first treatment. Dogs were randomized to treatment (six animals in each group) with six monthly oral doses of placebo, Simparica Trio, Heartgard Plus, or Interceptor Plus at their respective label doses. Microfilaria (MF) and antigen tests were conducted periodically, and efficacy was evaluated by necropsy for adult heartworms approximately 9 months after L3 inoculation.

**Results:**

Adult heartworms were recovered from all six placebo dogs, with a geometric mean of 35.5 worms (range, 23–48). Five of the six dogs treated with Simparica Trio were infected with a geometric mean of 1.0 worms (range, 0–3), and all remained MF-negative. All Heartgard Plus-treated dogs (six) were infected with a geometric mean of 32.5 worms (range, 22–38); five of these dogs were MF-positive at day 236. All Interceptor Plus-treated dogs (six) were infected with a geometric mean of 22.8 worms (range, 10–34); five of these dogs were MF-positive at day 236. The efficacy of six consecutive doses with Simparica Trio, Heartgard Plus, and Interceptor Plus against ZoeLA was 97.2, 8.5, and 35.9%, respectively. Adult worm counts for the Simparica Trio-treated group were significantly lower (*P* < 0.0001) than placebo control, Heartgard Plus, and Interceptor Plus-treated groups. Adult worm counts for Heartgard Plus and Interceptor Plus were not significantly different from placebo (*P* > 0.05).

**Conclusions:**

Simparica Trio prevented microfilaremia in all dogs and was highly effective (97.2%) and significantly better than either Heartgard Plus (8.5%) or Interceptor Plus (35.9%) in preventing the development of the ZoeLA ML-resistant heartworm strain when administered for six consecutive months in this comparative laboratory efficacy study.

**Graphical Abstract:**

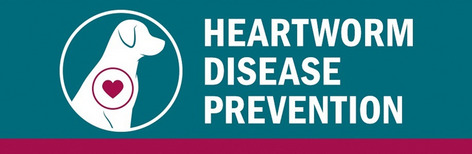

## Background

The filarial nematode *Dirofilaria immitis,* transmitted by mosquitoes, causes heartworm disease in dogs*.* Because *D. immitis* adults reside in the dogs’ pulmonary artery and heart, infections can cause life-threatening and severe cardiopulmonary disease [1].

With higher prevalence in warmer climates, canine heartworm disease is a global concern with more than 60 different species of mosquitoes capable of carrying this parasite [1]. The distribution of heartworm appears to be expanding globally, likely due to climate changes, that are allowing for the expansion of mosquito vectors in density, distribution, and seasonality, and increasing pet travel [2, 3].

The only drug widely available and highly effective in the prevention of canine heartworm disease belongs to the macrocyclic lactone (ML) class of anthelmintics [4]. Likely working in concert with the canine immune system, MLs kill the immature third (L3) and fourth (L4) larval stages, preventing the development of the adult *D. immitis* [5]. Failure to strictly comply with the approved label directions of ML heartworm preventives can result in a lack of efficacy (LOE) [4]. Recently, there has been growing concern, as at least 16 different field isolates collected from dogs in the United States, primarily concentrated in the Lower Mississippi River Valley (LMRV), have been identified as having genotypic and/or phenotypic resistance to MLs [4, 20]. When tested in preventive laboratory efficacy studies, some of these strains have demonstrated dramatic reductions in preventive efficacy for heartworm, making LOE due to ML resistance a growing concern [4, 7, 8, 21].

Moxidectin, an ML available in heartworm prevention products globally, demonstrates 100% efficacy in studies utilizing ML-susceptible *D. immitis* strains [4]. The pharmacokinetic properties of moxidectin along with its high degree of lipophilicity, which results in superior bioavailability, make it an attractive compound, either as a stand-alone or in a combination endectocide, for use as a heartworm preventive in the face of emerging resistance [11]. Against ML-resistant *D. immitis*, increasing the oral monthly moxidectin dosage from 3 µg/kg (the original approved oral dose for heartworm prevention) to 24 µg/kg of moxidectin and increasing the consecutive number of monthly administered doses has been shown to improve its efficacy [4, 10]. Moxidectin at 0.5 mg/kg is also effective in an extended-release injectable form against ML-resistant strains [10]. A new oral chewable tablet Simparica Trio^®^ utilizes this increased moxidectin dosage (ranging from 24 to 48 µg/kg) in combination with the ectoparasiticide sarolaner and the nematodicide pyrantel. While the oral point dosage of moxidectin alone at 24 µg/kg has been tested against ML-resistant heartworm strains [9], the commercial dosage of Simparica Trio has not been tested previously.

The study presented herein compared the preventive efficacy of six consecutive monthly doses of Simparica Trio^®^ to six consecutive monthly doses of Heartgard^®^ Plus (ivermectin/pyrantel) or Interceptor^®^ Plus (milbemycin oxime/praziquantel) administered orally against the confirmed ML-resistant *D. immitis* ZoeLA strain.

## Methods

### Design

This negative-controlled, comparative, masked, randomized laboratory study was conducted in accordance with the International Cooperation on Harmonisation of Technical Requirements for Registration of Veterinary Medicinal Products (VICH) guidelines [12, 13].

Treatment assignments were masked to personnel that made efficacy or safety assessments, including clinical observations, physical exams, L3 inoculation, treatment administration, and necropsy with adult *D. immitis* recovery.

The study design is summarized in Table 1.

**Table 1:** Efficacy of Simparica Trio^®^ against ML-resistant heartworms compared to Heartgard^®^ Plus or Interceptor^®^ Plus: study design

Group	Treatment	Oral dosage^b^	No. of dogs	Day of L3 *D. immitis* inoculation^a^	Days of treatment	Days of blood microfilariae and adult *D. immitis* antigen testing	Day of necropsy and adult *D. immitis* worm recovery
T01	Placebo control	na	6	-30	0, 30, 60, 90, 120, 150	-63, -35, 60, 180, 210, 236	241
T02	Simparica Trio	Label dosing	6				
T03	Heartgard Plus	Label dosing	6				
T04	Interceptor Plus	Label dosing	6				

^a^Each dog was inoculated with 50 infective L3 of *D. immitis* (ZoeLA isolate)

^b^Simparica Trio (minimum of 24 µg/kg moxidectin + 1.2 mg/kg sarolaner + 5 mg/kg pyrantel), Heartgard Plus (minimum of 6 µg/kg ivermectin + 5 mg/kg pyrantel) and Interceptor Plus (minimum of 500 µg/kg milbemycin) oxime + 5 mg/kg praziquantel) administered at their approved dosages according to their commercial label directions

### Animals

A total of 24 purpose-bred beagles, individually identified by ear tattoo or microchip, were used. Six dogs, both males and females, were in each treatment group (T01–T04). Dogs were 6 months old and negative for adult *D. immitis* antigen and blood microfilariae (MF) at the time of *D. immitis* L3 inoculation. On day 0, at the first treatment administration, body weight ranged from 7.2 to 11.3 kg. All dogs were assessed by a veterinarian as being in good health at the time of enrollment based on physical examination. No dog had received any monthly administered ML-containing product within 90 days prior to the start of the study or had ever received ProHeart^®^ 6 or ProHeart^®^ 12 (moxidectin; Zoetis, Parsippany, NJ, USA) during their lifetime.

All dogs were housed indoors within a mosquito-proof facility in compliance with accepted animal welfare guidance and legislation, with two dogs in each pen. Dogs were acclimated at the facility for 63 days prior to L3 inoculation and maintained under standard environmental conditions with environmental enrichment and social interactions provided. Dogs had ad libitum access to water and were fed an appropriate canine commercial maintenance diet. Throughout the study, dogs were observed at least once daily for general health.

### *Dirofilaria immitis* strain

The *D. immitis* ZoeLA strain used in this study was derived from an isolate collected from a naturally infected dog in Louisiana in June of 2013. The isolate was validated as an infective strain through positive heartworm antigen test results, the diagnosis of circulating MF, and adult worm recovery in recipient animals following inoculation of L3 that were derived directly from MF obtained from the original field case. This *D. immitis* strain has been demonstrated to be resistant to MLs [4, 8, 10].

### *Dirofilaria immitis* L3 inoculations

Using previously described techniques, the L3 were harvested from infected *Aedes aegypti* mosquitoes [15] that were reared and maintained at Zoetis (Kalamazoo, MI, USA) for use in inoculations.

Fifty viable L3 were administered to each dog by subcutaneous injection in the inguinal region on day 0, 30 days prior to the first treatment dosing.

### Randomization and treatments

Animals were randomly assigned to pens and treatments using a randomized complete block design based on day -3 body weights and pen location. Pairs of animals of the same sex were formed after sorting animals by body weight within sex. Pairs were then ranked by average body weight across sex, and blocks of four pairs were formed. Pens were assigned to blocks so that four neighboring pens occurred in each block. Pairs within a block were then randomized to treatment groups and to pens within blocks. The experimental unit for treatment was the pen.

Treatments were administered to all groups on days 0, 30, 60, 90, 120, and 150. Dogs in the placebo group (T01) were administered an empty hydroxypropyl methylcellulose (HPMC) capsule on days 0, 30, 60, 90, 120, and 150. The respective test materials were administered to dogs in the Simparica Trio (T02), Heartgard Plus (T03), or Interceptor Plus (T04) groups.

Simparica Trio was provided by Zoetis in commercial packaging, while Interceptor Plus chewable tablets and Heartgard Plus chewables were obtained from a commercial supplier. Treatments were administered according to their approved commercial dosing instructions, which resulted in dogs being treated with Simparica Trio receiving 24–44.9 µg/kg moxidectin, with Heartgard Plus receiving 6.2–11.8 µg/kg ivermectin, and with Interceptor Plus receiving 0.5–1.0 mg/kg milbemycin oxime, with the exception of three doses. Two dogs in the Simparica Trio group and one dog in the Heartgard Plus group were inadvertently underdosed only on day 60. These dogs received 50% of the intended commercial dose and 96.3–99.2% of the minimum dose (expanding the dose range to 23.1–44.9 µg/kg moxidectin) and 93.3% of the minimum dose (expanding the dose range to 5.6–11.8 µg/kg ivermectin) per product labeling for the Simparica Trio-treated dogs and Heartgard Plus-treated dog, respectively.

Within 3 days prior to each treatment administration, body weights were obtained to use for dose calculations. Prior to dosing, feed was withheld overnight and until at least 4 h after treatment administration. All doses were administered orally. After dosing, each dog was observed for several minutes for evidence that treatment was swallowed, and approximately 2 h after the dosing event for evidence of regurgitation or emesis. Dogs were housed individually for 1 week post-dose for observation.

### Blood microfilariae and adult *D. immitis* antigen testing

On days -63, -35, 60, 180, 210, and 236, blood was collected from each dog to examine for blood MF and adult *D. immitis* antigen testing. Blood collected on days -63, -35, and 60 was tested to reveal *D. immitis* infection pre-existing prior to experimental inoculation, while testing of blood obtained on days 180, 210, and 236 was conducted to reveal *D. immitis* infection from the experimental L3 inoculation.

The DiroCHEK^®^ Heartworm Antigen Test Kit (Zoetis), a commercially available test kit, was used for *D. immitis* antigen detection, and blood MF examination used the previously described modified Knott’s procedure [5]. All samples negative for *D. immitis* antigen from day 236 were heat-treated and the antigen testing was repeated [6].

### Health observations

Twice daily, general health observations were made for each dog except on dosing days and on days that physical examinations were performed (63 and 32 days prior to L3 inoculation and just prior to euthanasia). Prior to and at 1, 3, 6, and 24 h after each dose administration directed clinical observations were made for each dog. Dogs were housed individually for 1 week after treatments to facilitate health observations.

### Necropsy and adult *D. immitis* worm recovery

On day 241 all dogs were humanely euthanized with a pentobarbital euthanasia solution administered intravenously according to the approved label directions with 1 ml of heparin added to prevent blood coagulation in the heart and lungs during dissection for worm recovery. In order to locate adult *D. immitis,* peritoneal and pleural cavities were examined, cranial and caudal vena cavae were clamped, and cardiopulmonary organs were removed. Examination during and after dissection of the precava, right ventricle, right atrium, and pulmonary arteries (including those with intrapulmonary coursing) was conducted. All adult worms recovered were classified as female or male and as either dead (abnormal in motility and appearance) or alive (all worms not fitting the dead definition) as previously described [16]. Order of euthanasia and necropsy for dogs was randomly assigned.

### Statistical analysis

The primary endpoint was the total (live + dead) worm count. The pen was the experimental unit. A general linear mixed model with fixed effect of treatment and random effects of block, block by treatment interaction and error was used to analyze total worm count (SAS 9.4, Cary, NC, USA). Worm counts were natural log-transformed prior to analysis [ln(count + 1)] as a remedial measure for normality and homoscedasticity assumptions. Hypothesis testing was at the two-sided 0.05 level of significance.

Percent efficacy relative to negative control was calculated using geometric means (back-transformed least-squares mean [LSM]) based on the formula [(C − T)/C] × 100, where T is the mean worm count for the treated group and C is the mean worm count for the negative control group. T was calculated for each treatment group individually for T02, T03, and T04.

## Results

### Dosing

Dosing was unremarkable: no capsules, chewable tablets, or chews were expelled during or post-treatment. During treatment on day 0, one Heartgard Plus-treated dog was observed to vomit a small amount of liquid approximately 1 h post-dose with no evidence of an expelled chewable tablet. With that exception, no emesis of capsules, chewable tablets, or chews was observed after treatment.

### Health observations

During the study, there were neither treatment-related adverse reactions nor mortalities. Abnormal health events were minor and those commonly observed in laboratory beagles, such as gastrointestinal, dermatologic, otic, and musculoskeletal abnormalities. The incidences of abnormal health events were similar between treatment groups.

### Parasitological parameters

**Fig. 1:**
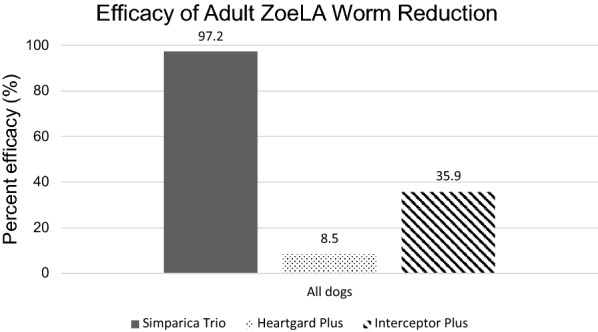
Efficacy of Simparica^®^ Trio against ML-resistant heartworm strain ZoeLA compared to Heartgard^®^ Plus or Interceptor^®^ Plus

Results of adult *D. immitis* antigen and blood MF testing results are summarized in Table 2. Summarized in Table 3 are adult worm counts, efficacies relative to the negative control, and statistical comparisons. Overall adult heartworm efficacy results are summarized in Fig. 1.

**Table 2:** Adult *D. immitis* antigen and microfilariae test results (ZoeLA strain)

Treatment group	Days of treatment	Individual	Day-35		Day 60		Day 180		Day 210		Day 236	
			Ag	MF	Ag	MF	Ag	MF	Ag	MF	Ag	MF
Placebo	0, 30, 60, 90, 120, 150	1	−	−	−	−	+	+	+	+	+	+
		2	−	−	−	−	+	+	+	+	+	+
		3	−	−	−	−	+	+	+	+	+	+
		4	−	−	−	−	+	+	+	+	+	+
		5	−	−	−	−	+	+	+	+	+	+
		6	−	−	−	−	+	+	+	+	+	+
Simparica Trio^®^^a^	0, 30, 60, 90, 120, 150	1	−	−	−	−	−	−	−	−	[−]	−
		2‡	−	−	−	−	+	−	+	−	+	−
		3	−	−	−	−	−	−	−	−	−	−
		4‡	−	−	−	−	−	−	−	−	[−]	−
		5	−	−	−	−	−	−	−	−	[−]	−
		6	−	−	−	−	−	−	+	−	+	−
Heartgard^®^ Plus^b^	0, 30, 60, 90, 120, 150	1	−	−	−	−	+	+	+	+	+	+
		2	−	−	−	−	+	+	+	+	+	+
		3	−	−	−	−	+	+	+	+	+	+
		4	−	−	−	−	+	+	+	+	+	+
		5	−	−	−	−	+	+	+	+	+	+
		6	−	−	−	−	+	+	+	+	+	−
Interceptor^®^ Plus^c^	0, 30, 60, 90, 120, 150	1	−	−	−	−	+	+	+	+	+	+
		2	−	−	−	−	+	+	+	+	+	+
		3	−	−	−	−	+	+	+	+	+	+
		4	−	−	−	−	+	+	+	+	+	+
		5	−	−	−	−	+	+	+	+	+	+
		6	−	−	−	−	+	+	+	−	+	−

^a^Simparica Trio (24 µg/kg moxidectin + 1.2 mg/kg sarolaner + 5 mg/kg pyrantel) was administered according to commercial label direction, which resulted in the administration of {23.1‡}25.0–44.9 µg/kg of moxidectin

^b^Heartgard Plus (6 µg/kg ivermectin + 5 mg/kg pyrantel) administered according to commercial label directions, which resulted in administration of {5.62‡} 6.2–11.8 µg/kg of ivermectin

^c^Interceptor Plus (500 µg/kg milbemycin oxime + 5 mg/kg praziquantel) administered according to commercial label directions, which resulted in administration of 0.5–1.0 mg/kg milbemycin oxime

‡ Designates individuals that were mis-dosed and altered dosing outside of commercial labeling that resulted

Brackets [] indicate samples heat-treated and adult heartworm antigen tests conducted again that were negative after heat treatment

Ag, adult *D. immitis* antigen test; MF, *D. immitis* microfilariae test; Min, minimum; − negative, + positive

**Table 3:** Efficacy of oral Simparica Trio^®^ compared to Heartgard^®^ Plus or Interceptor^®^ Plus (ZoeLA strain)

Treatment	Oral ML dosage	Days of treatment	No. of infected dogs^a^	Adult *D. immitis* worm counts		Efficacy compared to negative control^c^
				Individual worm counts	Geometric mean^b^	% Reduction
Negative control	na	0, 30, 60, 90, 120, 150	6 of 6	23, 34, 36, 37, 40, 48	35.5 ^d^	na
Simparica Trio^f^	Min. 24 µg/kg moxidectin	0, 30, 60, 90, 120, 150	5 of 6	0, 1, 1, 1, 1‡, 3‡	1.0^e^	97.2
Heartgard plus^g^	Min. of 6 µg/kg ivermectin	0, 30, 60, 90, 120, 150	6 of 6	22, 33, 33, 34, 38, 3‡‡, 8	32.5^d^	8.5
Interceptor plus	Min. of 500 µg/kg milbemycin oxime	0, 30, 60, 90, 120, 150	6 of 6	10, 12, 32, 32, 32, 34	22.8^d^	35.9

^a^Each dog was inoculated with 50 infective ZoeLA L3 of *D. immitis* on day −30

^b^Geometric mean counts with the same superscript letters (d–e) are not significantly different (*P* > 0.05)

^c^All dogs were necropsied for recovery of adult *D. immitis* on day 241 (271 days post-inoculation)

^f^Analysis excluding underdosed dogs removed two dogs from this group with individual worm counts designated (‡); three of the four dogs remaining were infected with a geometric mean of 0.8 worms and reduction of 98.1%

^g^Analysis excluding underdosed dogs removed one dog from this group with an individual worm count designated (‡‡); five of the five dogs remaining were infected with a geometric mean of 31.5 worms and reduction of 11.3%

#### Microfilaria and adult heartworm antigen testing

All but one (5 of 6) of the Simparica Trio-treated dogs were negative for *D. immitis* antigen and MF on day 180, while the remaining dog was positive for the antigen but negative for MF for this day. Two dogs were positive for *D. immitis* antigen but not MF on days 210 and 236. Four Simparica Trio-treated dogs were negative for both antigen and MF on days 210 and 236. All six Heartgard Plus-treated dogs were positive for *D. immitis* antigen and MF on days 180, 210, and 236, except for one dog that was negative for MF on day 236. All six Interceptor Plus-treated dogs were positive for *D. immitis* antigen and MF on day 180, and five of the six dogs were MF-positive on days 210 and 236. The remaining dog was positive for antigen but was negative for MF on these days. Details are provided in Table 2.

#### Adult heartworm (ZoeLA strain) counts

All six placebo dogs had heartworms, with a geometric mean of 35.5 worms (range, 23–48). Five of the six dogs treated with Simparica Trio had heartworms (one had none), with a geometric mean of 1.0 worms (range, 0–3). All six dogs treated with Heartgard Plus had heartworms with a geometric mean of 32.5 worms (range, 22–38), and all six dogs treated with Interceptor Plus had heartworms with a geometric mean of 22.8 worms (range, 10–34).

Geometric mean worm counts for the Simparica Trio group were significantly lower than those for the negative control group (*t*_(6)_ = 13.30, *P* < 0.0001). Meanwhile, geometric mean worm counts for Interceptor Plus and Heartgard Plus groups were not significantly different from the placebo group (0.39 ≤ *t*_(6)_ ≤ 1.97, *P* ≥ 0.0965), and were not significantly different from each other (*t*_(6)_ = 1.57, *P* = 0.1664). The geometric mean worm counts for the Simparica Trio group were significantly lower than the groups treated with either Heartgard Plus or Interceptor Plus (−12.91 ≤ *t*_(6)_ ≤  −11.33, *P* < 0.0001).

Preventive efficacies relative to negative control were 97.2% for the group treated with six monthly doses of Simparica Trio, 8.5% for the group treated with Heartgard Plus, and 35.9% for the group treated with Interceptor Plus with all dogs included. Preventive efficacies were 98.1%, 11.3%, and 35.9%, respectively, if the dogs mis-dosed once were excluded, with no difference in statistical power.

## Discussion

Given the serious and potentially deadly nature of heartworm disease, the goal of prevention focuses appropriately on 100% preventive efficacy of the treatment. Pulmonary arterial disease is ubiquitous in infected dogs, with adult worms beginning to cause traumatic arterial damage days after reaching the caudal pulmonary arteries [17, 18]. Although many infected dogs show no clinical signs, infections with higher worm burdens tend to result in more significant clinical disease [17], including congestive heart failure due to pulmonary hypertension [19, 24] and increased risk of fatal caval syndrome [1, 5].

The most potent ML against heartworm appears to be moxidectin based on reported efficacy data on the prevention of the development of *D. immitis* in dogs [4, 14]. Persistence and greater distribution of moxidectin are apparent consequences of its longer elimination half-life and larger distribution volume in host tissues, including adipose tissue where it is likely active against migrating *D. immitis* larvae, compared to ivermectin and milbemycin [11]. Oral moxidectin (24 µg/kg) as a single dose, alone and in combination as Simparica Trio (with sarolaner and pyrantel) has demonstrated 100% efficacy against ML susceptible field strains of *D. immitis* [20] and significantly better efficacy than placebo or positive controls (Heartgard Plus and Interceptor Plus) against known ML-resistant strains (ZoeLA and JYD-34) when administered either 4 or 6 months consecutively [9].

Likewise, in field studies, this difference between moxidectin and ivermectin efficacy has been borne out. In a large clinical field study with over 240 veterinary patients enrolled from clinics where heartworm disease is endemic, including 10% of patients from multiple clinics in the LMRV region where ML resistance occurs, 11 monthly doses of moxidectin (24 µg/kg) administered in combination with sarolaner and pyrantel were completely effective in preventing the development of heartworms [20]. Another field study involving 19 clinics in heartworm endemic areas, including in the LMRV, used ProHeart^®^ 12 (Zoetis), an extended-release moxidectin formulation (0.5 mg/kg), provided 100% preventive efficacy in more than 230 client-owned dogs [23]. The positive control in both studies, Heartgard Plus, failed to provide complete protection, with two of 117 (1.7%) in the former and four of 218 (1.8%) in the latter study becoming positive. Of those, one of 117 (0.9%) and three of 218 (1.4%) developed patent infections, respectively. The development of patent infections in dogs from two separate studies under natural *D. immitis* exposure while receiving Heartgard Plus with confirmed compliance, strongly suggests that these four dogs were infected with ML-resistant heartworm strains [21–23]. These studies support the use of moxidectin in new heartworm preventive products in the face of emerging and potential spread of ML heartworm resistance.

In the present study, six consecutive monthly doses of Simparica Trio provided 97.2% efficacy relative to the negative control, compared to the 8.5% efficacy provided by Heartgard Plus and the 35.9% efficacy provided by Interceptor Plus (Fig. 1). Six monthly doses of Simparica Trio also resulted in significantly fewer (−12.91 ≤ *t*_(6)_ ≤ −11.33, *P* < 0.0001) adult *D. immitis* in dogs compared to Heartgard Plus or Interceptor Plus against the known ML-resistant ZoeLA *D. immitis* strain.

To assess the impact of the day 60 underdosing in the two Simparica Trio-treated dogs and the one Heartgard Plus-treated dog on overall results, a separate analysis was conducted excluding data from these dogs. These results are presented in Table 3. Results were similar to the analysis with data from these dogs included, in which the geometric mean worm count for the Simparica Trio-treated group was 0.8, with a 98.1% reduction in worm count compared to the negative control group (*P* < 0.0001). For the Heartgard Plus-treated group, the geometric worm count was 31.5, with an 11.3% reduction in worm count compared to negative control (*P* = 0.6184).

The mis-dosing of the dogs on day 60 provides parallels to the real world, where compliance with label instructions is imperfect. These dogs received doses per label for 2 months, were underdosed for 1 month, and then treated per label dose for 3 more months, which could occur in a clinical setting, such as a dog temporarily gaining weight that could similarly be mis-dosed for 1 month, or a dog that gained weight and was out of the labeled dose before the dog was reassessed by a veterinarian and prescribed the appropriate larger dose of heartworm preventive. With or without perfect label adherence in this study, Heartgard Plus and Interceptor Plus lacked a significant reduction in worm burdens compared to placebo with the range of worms unaffected by it (6 to 37 worms). In contrast, treatment with Simparica Trio with or without perfect label adherence resulted in no worms recovered at necropsy for one dog and low worm burdens for the other five (range 1–3). None of the Simparica Trio-treated dogs developed MF: fewer dogs positive for MF also reduces the opportunity for the spread of ML-resistant *D. immitis* by minimizing the reservoir for mosquitoes.

The preventive efficacies provided by Simparica Trio against the ML-resistant *D. immitis* strain evaluated in the present study support the outcomes from previous studies [4, 10]. Comparisons of data from the present and previous studies are presented in Table 4. Against ML-resistant strains, three [4], four [4, 9], and six [9] monthly doses of 24 µg/kg moxidectin resulted in significantly fewer adult worms (*P* < 0.0001) and ≥ 98.8%, ≥ 95.9%, and ≥ 96.1% efficacy relative to placebo control, respectively (see Table 4)*.* The current recommendation to prevent heartworm disease by the American Heartworm Society is continuous year-round administration of preventive drugs [5].

**Table 4:** Efficacy of oral moxidectin against ML-resistant heartworm strains compared to Simparica Trio^®^, Heartgard^®^ Plus or Interceptor^®^ Plus: summary

Oral treatment^a^	No. of monthly treatments^b^	ZoeLA				JYD-34			
		No. of infected dogs^c^	Adult *D. immitis* worm counts		Efficacy compared to negative control (%)	No. of infected dogs^c^	Adult *D. immitis* worm counts		Efficacy compared to negative control (%)
			Range	Geometric mean			Range	Geometric mean	
Moxidectin	1	Not tested				8 of 8	7–29	16.8	53.2^d^
Moxidectin	3	1 of 5	0–1	0.1	99.5^d^	1 of 5	0–2	0.2	98.8^d^
Moxidectin	4	5 of 6	0–3	1.1	96.8^e^	5 of 6	0–4	1.3	95.9^e^
Moxidectin	6	5 of 6	0–4	1.4	96.1^e^	2 of 6	0–1	0.2	99.3^e^
Simparica Trio	6	5 of 6	0–3	1.0	97.2^ g^	na	na	na	na
Heartgard Plus	6	6 of 6 6 of 6	25–36 22–38	29.0 32.5	18.7^e^ 8.5^ g^	6 of 6 6 of 6 6 of 6	6–18 12–29 19–34	11.9 21.7 26.8	63.9^e^ 37.7^f^ 10.5 ^f^
Interceptor Plus	6	6 of 6 6 of 6	16–37 10–34	28.1 22.8	21.2^e^ 35.9^ g^	6 of 6 6 of 6 6 of 6	10–22 14–37 13–35	14.9 22.7 25.5	54.6^e^ 34.9^f^ 14.6^f^

^a^Moxidectin administered at exact 24 µg/kg dosage. Simparica Trio (24 µg/kg moxidectin + 1.2 mg/kg sarolaner + 5 mg/kg pyrantel), Heartgard Plus (6 µg/kg ivermectin + 5 mg/kg pyrantel), and Interceptor Plus (500 µg/kg milbemycin oxime + 5 mg/kg praziquantel) administered according to commercial label directions, which resulted in administration of 24 to 48 µg/kg moxidectin, 6.2–15.5 µg/kg of ivermectin and 0.5 to 1.2 mg/kg milbemycin oxime

^b^Treatment administration days: one monthly treatment: day 0 [4]; three monthly treatments: days 0, 28, and 56 [4]; four monthly treatments: days 0, 30, and 60 [9], six monthly treatments: days 0, 30, 60, 90, 120, and 150 [9, 10, studies from this paper], and for one study, six monthly treatments were provided prior to and after inoculation on day 165 (treatments on days 0, 30, 60, 90, 120, 150, 180, 210, 240, 270, 300, 330) [10]

^c^Each dog inoculated with 50 *D. immitis* L3 on day -30, with adult heartworm recovery at necropsy 133–273 days after inoculation

^d^Data reference: McTier et al. [4]

^e^Data reference: Kryda et al. [9]

^f^Data reference: McTier et al. [10]

^g^Data reference: this paper

Simparica Trio and 24 µg/kg of moxidectin alone provide very high efficacy (> 96%) in preventing the development of heartworms in dogs after 3–6 consecutive monthly doses. Some dogs were completely free of heartworms, and many dogs had a single worm with a geometric mean range of 0.1–1.4 heartworms (Table 4). Meanwhile, dogs treated with six monthly doses of Heartgard Plus and Interceptor Plus were all infected with multiple worms with geometric mean ranges of 8.5–18.7 and 21.2–35.9 worms, respectively. Since higher worm burdens can be correlated with more severe symptomatology, including pulmonary hypertension [5, 17, 24], this difference in worm burden, especially against ML-resistant heartworm strains, could engender a substantial clinical benefit over time for those dogs treated with Simparica Trio.

It is estimated that a meager 30% of dogs in the USA are on an ML preventive, so despite MLs effectiveness in the prevention of heartworm with appropriate dosing, dogs are at risk of developing heartworm disease. The cause for LOE is most commonly a failure to administer the product regularly according to label instructions [25]. Furthermore, a number of multiple ML-resistant *D. immitis* strains in the field have been confirmed [4]. As the understanding of the geographical range of ML-resistant heartworm strains is incomplete, as well as the factors, which could potentially facilitate future spread of these resistant strains, e.g., travel of dogs, use of preventive products broadly across the USA, which provide the most potent efficacy against all heartworm strains seems prudent.

Collectively, data from the present laboratory study and those from earlier laboratory and field studies demonstrate that monthly administration of oral Simparica Trio (minimum of 24 µg/kg moxidectin) provides robust heartworm prevention against *D. immitis* strains to which most dogs in the USA will likely be exposed, including those strains such as ZoeLA that have been confirmed to be ML-resistant.

## Conclusions

In this laboratory study, designed to assess preventive efficacy against the ML-resistant *D. immitis* ZoeLA strain, Simparica Trio administered for six consecutive months provided 97.2% reduction in ML-resistant adult *D. immitis* relative to negative controls. No Simparica Trio-treated dogs developed MF. Additionally, Simparica Trio was significantly more effective than either Heartgard Plus or Interceptor Plus administered for 6 months at their approved label dosages in preventing the development of the ZoeLA ML-resistant heartworm strain.

## Data Availability

Data supporting the conclusions of this article are included within the article.

## Abbreviations

HPMC: Hydroxypropyl methylcellulose
JYD-34: *Dirofilaria immitis* strain JYD-34
L3: Third-stage larvae
LMRV: Lower Mississippi River Valley
LOE: Lack of efficacy
LSM: Least-squares mean
ML: Macrocyclic lactone
ZoeLA: *Dirofilaria immitis* strain ZoeLA-2013
